# Depression diagnosis and treatment amongst multimorbid patients: a thematic analysis

**DOI:** 10.1186/1471-2296-15-124

**Published:** 2014-06-19

**Authors:** Melinda N Stanners, Christopher A Barton, Sepehr Shakib, Helen R Winefield

**Affiliations:** 1 Discipline of General Practice, Flinders University, Bedford Park, South Australia, Australia; 2 Social Health Sciences Unit, Flinders University, Bedford Park, South Australia, Australia; 3 Department of Clinical Pharmacology, Royal Adelaide Hospital, Adelaide, South Australia, Australia; 4 School of Psychology, University of Adelaide, Adelaide, South Australia, Australia

**Keywords:** Depression, Multimorbidity, Chronic illness and disease, Patient experiences, Qualitative interviews

## Abstract

**Background:**

We explored experiences of depression diagnosis and treatment amongst multimorbid patients referred to a metropolitan multidisciplinary outpatient clinic to identify commonalities across this patient group.

**Methods:**

Patients with two or more chronic conditions and a diagnosis of depression participated in semi-structured interviews that were digitally recorded and transcribed. Thematic analysis was performed on the transcriptions.

**Results:**

Multimorbid patients attributed depressive symptoms to the loss of ‘normal’ roles and functionality and struggled to reconcile the depression diagnosis with their sense of identity. Beliefs about themselves and depression affected their receptivity to diagnosis and intervention strategies. These included prescribed interventions, such as psychotherapy or pharmacotherapy, and patient-developed strategies.

**Conclusions:**

Functional and social role losses present a clear context in which GPs should raise the subject of mood, with the situational attribution of depression suggesting that psychotherapy, which is rarely offered, should be prioritised in these circumstances.

## Background

Depression has a substantive negative impact on quality of life. Research over the last twenty years suggests that depression is under-diagnosed and under-treated amongst older people in Western nations [1-4], and the literature suggests that general practitioners (GPs) are less successful at identifying depression in older patients than younger patients [5].

Differences between patient and clinician perceptions of depression might contribute to poor diagnosis [6], as patients may resist formal treatment where GP and patient care priorities clash [7]; consequently, GPs may be reluctant to broach the subject of depression unless the patient does [8]. Age-related effects on the manifestation of depressive symptoms have been noted, with older adults more likely to report somatic symptoms than complain about emotional distress [9]. As the risk of developing one or more chronic illness increases with age [8,10], the presence of multiple chronic illnesses may also affect the manifestation of depression and increase the risk of normalisation of depression [11].

Previous studies exploring patient experiences of illness and depression in specific patient groups have identified themes such as the burden of uncertainty amongst gynaecological patients [12] and chronic heart failure patients [13] as contributory to depression, and guilt, perceived stigma, duty to be well, and shame regarding self-regulation of mood amongst depressed adults [14,15], but no research to date has explored the experience of depression onset, diagnosis and treatment in patients with multiple chronic conditions [16].

Irrespective of age, gender or specific disease, the risk of depression increases with each chronic condition acquired [10]. The presence of two or more chronic conditions is referred to as ‘multimorbidity’ [17]. Developing tools, methods and markers for detecting depression in multimorbid patients will become more topical over the next forty years, as increased longevity will increase the number of people experiencing multiple chronic conditions [18]; consequently, the identification of experiences that occur in common across multimorbid patients diagnosed with depression could assist GPs in discerning when to engage in conversations about mood, and inform diagnostic and treatment practices.

To this end, we aimed to explore the experiences of multimorbid patients in the development, diagnosis and treatment of depression to identify any common experiences and themes that may occur across a heterogenous and complex patient group.

## Methods

This qualitative study was conducted within a constructivist epistemology, in which concepts of reality are considered to be socially constructed, using an interpretivist theoretical perspective, where the exploration of patient narratives are acknowledged to be interpreted by the researcher [19]. Corbin and Strauss’ strategies for qualitative research [20] were used to guide data collection and analysis.

Participants were recruited from amongst patients who were referred to a multidisciplinary clinic at a metropolitan hospital in Adelaide for the management of multiple chronic conditions between January and June 2011. Consistent with the definition most commonly used in multimorbidity literature [17], patients with two or more chronic conditions and a diagnosis of depression were invited to participate. Depression was diagnosed prior to referral to the clinic; consequently a diagnosis of depression was derived from GP referral letters and discharge summaries prior to the patient’s attendance at the multidisciplinary clinic and was not treated as part of care provided by clinic doctors. Patients were excluded if they suffered cognitive impairment, or their language skills did not support an independent conversation in English. Because of the unique influences on and higher prevalence of depression in residential care facilities, residents in such facilities were also excluded.

Thirty-five patients with a diagnosis of depression attended the clinic over the six month period of recruitment. Six were excluded due to language difficulties; four refused; three agreed but on further discussion were not eligible, and one agreed but subsequently could not be contacted. Recruitment opportunities were lost where patients were not approached because of competing clinic priorities or patient non-attendance at their clinic appointment (N = 9). During recruitment, clinic nurses explained the aims of the study, gave patients an information sheet, and obtained informed consent for them to be contacted by the interviewer (MS) to arrange an interview. Of those who agreed to participate, one could not be contacted to arrange an interview. In accordance with the conventions of thematic analysis, recruitment ceased when no new information emerged from the interviews [20].

The semi-structured interview guide contained the following prompts:

• Tell me about the first time you experienced depression.

• Who did you talk to about how you were feeling? Family/Friends/GP

• What does the word ‘depression’ mean to you?

• I’d like you to think back to when you were first diagnosed with depression. How was it eventually diagnosed? Tell me about that.

• Tell me about what treatment was suggested. How did you feel about the options that they recommended? What affected your decision about whether or not to follow their recommendation?

• Did anything prevent you from accessing or continuing to access treatment?

• Did you receive any follow-up care from your GP?

• What other support or information would you have liked to have received?

Three interviews occurred at the hospital, seven occurred at patients’ homes, and two occurred over the telephone. Seven women and four men were interviewed alone; one man was assisted by his wife during the interview. Ages ranged from 48 to 86 years of age (median = 64.5). Interviews ranged from 17 to 68 minutes in length (average = 46.7 minutes), and were digitally recorded as audio files and transcribed verbatim using transcription software program Express Scribe v 5.13 [21]. Audio recording failed during one interview, and thematic analysis was performed using the researcher’s written notes. The transcripts were entered into NVivo 9 [22] to assist data management and facilitate analysis.

Inductive thematic analysis, whereby themes are generated from the data as opposed to a pre-existing thematic framework, was performed concurrently with interviews until thematic saturation was reached, in accordance with the methods described by Corbin and Strauss [20]. Journal entries and memos were included in the analysis. Thematic analysis took an iterative approach, whereby as new themes were identified and added to the thematic framework, earlier transcripts were recoded [20]. The coding structure was confirmed by an experienced qualitative researcher (CB). Ethics approval was granted by the Royal Adelaide Hospital Research Ethics Committee.

## Results

Thematic saturation was reached after ten interviews; two confirmatory interviews were conducted and no new themes arose from those interviews [20]. The interviews elicited descriptions of multimorbid participant contexts for developing depression, and their experiences of the detection and management of depression. The core category, ‘Everyone’s different, everyone’s the same’ is an apropos description of the diverse stories that generated a number of common themes, suggesting a strong common experiential trend. The results of the thematic analysis are described below.

### Depression development: the loss of identity

All participants described depression as developing subsequent to a life event that resulted in the loss of their identity. The most frequently endorsed event was onset or exacerbation of illness (n = 7 participants) where increased symptom burden promoted functional losses that prevented participants from performing their work or family roles. Participants described the interdependent losses of function and roles, and subsequent impact on their sense of identity, as causative of depression.

“I started to get depressed because I couldn’t do the things that I was always doing. You know, looking after my family, cooking, things like that because I was told I had to get off my feet, I wasn’t allowed to walk… So my life sort of just, you know, from being a normal mother, wife and that, running around and doing my thing, to doing nothing at all”.

(Female aged 59, 10 chronic conditions)

Participant stories also included unwanted gains. Many described accompanying burdens such as pain, fear of the unknown and family tension. Pain was the only symptom of physical illness that multimorbid participants reported as a perceived contributor to depression, with several describing the development of depression as being embedded in their ongoing struggle with pain. “Every day’s so hard, you know, to cope, well that’s with-- the [morphine] pump’s good, but all it does is take the edge off, you still have severe, you still have severe pain”. (Male aged 49, 7 chronic conditions)

### Depression diagnosis: I’m not ‘depressed’

Multimorbid patient beliefs about depression and their personal identity affected their receptivity to the diagnosis. The majority of participants (n = 9) reported being diagnosed by their GP, with one diagnosed following GP referral to a psychiatrist, and two diagnosed during hospital admission for physical illness. Whereas a small number of participants had anticipated the diagnosis and were receptive to the interventions recommended by their clinician, most of these participants were surprised by the diagnosis and reported rejecting the diagnosis at the time of delivery. Multimorbid participants who rejected the diagnosis described incongruence between their beliefs about depression and their beliefs about their own identity. “I guess initially it sort of shocked me, because I thought that I wasn’t sort of in that category…” (Male aged 65, 10 chronic conditions). “…I’m very strong person, and I don’t allow myself, you know, to be how shall I say, overcome, you know, by emotions… Well, I was surprised”. (Female aged 80, 8 chronic conditions)

When probed about their conceptualization of depression, participants described cognitive and emotional symptoms. One participant described depression as a response to the behaviour of other people, and another described it as a consequence of poor response management. Some participants interpreted their symptoms through their beliefs about depression, perceiving themselves as anxious but not depressed, or believing their emotional symptoms to be subclinical to a diagnosis of depression. One participant drew her interpretation of depression from television advertisements for a national mental health initiative (BeyondBlue), stating that because her symptoms were not identical, the diagnosis was inaccurate. “See, on TV now there’s adverts about depression with young people, and that type of thing? So I haven’t felt like that, just maybe down for a little while”. (Female aged 61, 5 chronic conditions)

A subtheme of perceived justification for depression emerged. Several participants (N = 3) expressed the belief that they did not have the right to feel depressed, despite the impact of their multiple chronic conditions on their physical and social functioning. These participants devalued their distress by minimizing the impact of their physical symptoms in comparison to those of others, and stating that they “had no reasons for being in this [state]” (Male aged 62, 5 chronic conditions). Conversely, the belief that physical illness justified depressive symptoms and nullified the veracity of the depression diagnosis was reported (N = 2). “But because I know why I’m like that and I feel that it’s justified, I don’t think that I’m clinically depressed, do you know what I mean? Because I feel that my condition justifies my feelings”. (Female aged 75, 12 chronic conditions)

Stigma regarding depression might have influenced rejection of the diagnosis more frequently than reported. Whereas many participants described rejecting the diagnosis, only one reported overt stigma in her reaction to the diagnosis.

“…here I go, I’m nutsville.’ And I didn’t agree with it. At first I fought the idea of being on antidepressants, but then realized I couldn’t cope the way I was going, and then went on antidepressants. . . . But at first I thought, oh no, here I go, I’m a nutcase, nobody’s going to take me seriously, and you know, it was embarrassing”.

(Female aged 48, 8 chronic conditions)

Stigma was observed in non-explicit verbal cues, such as where an initially verbose participant became defensive and refused to discuss depression, and another expressed willingness to help, but consistently avoided answering questions about depression. Stigma was also reflected in non-verbal cues during interviews, such as defensive body language (leaning back, crossing arms, and looking down), tone, and facial expressions, and in the use of words that devalued their emotional experience or distanced them from the diagnosis. “So there’s highs and lows, but I wouldn’t say, like, great depression”. (Female aged 61, 5 chronic conditions).

### Depression interventions: prescription, self-efficacy and coping

Multimorbid patients reported that GPs offered interventions within the medical framework, such as pharmacotherapy and occasionally psychotherapy. Acceptance of GP treatment recommendations was strongly influenced by beliefs about the interventions and anticipated outcomes, the cause of their depression, and their sense of self-efficacy. Whereas one participant admitted that he had benefitted from psychotherapy, for example, he perceived it as ultimately unsuccessful because the benefit was temporary. “Yeah yeah, it was helpful, unfortunately it doesn’t last forever. . . You get it off your chest. But as I said it doesn’t last forever”. (Male aged 65, 10 chronic conditions)

Beliefs about antidepressant safety and efficacy stemmed from observing another person’s experience, perceived risk of side effects, and the threat of disruption to their existing medication regimen. The majority of participants described trialling at least one anti-depressant medication, which either changed or reinforced their pre-treatment beliefs. Experiencing side effects led to accepting different medication, or refusing medication. Refusal occurred particularly where participants perceived their depression as situational and not medical. “I was pretty down on myself in all respects, and couldn’t understand how I fucked up my life, excuse me and I just didn’t think a tablet could take that away”. (Male aged 62, 5 chronic conditions)

Participants also described engaging in activities outside of the medical framework to alleviate negative mood and maintain a positive sense of identity, either in addition to or instead of prescribed interventions. Empowering activities, such as exercise and pet ownership, generated a sense of control and positive beliefs about the participant’s personal role in mood management. Disempowering activities were also reported, such as alcoholism, withdrawal and wishful thinking. Coping strategies, such as thinking of themselves as lucky and accepting their multimorbidities, were also described.

## Discussion

The stories of diagnosis and treatment of depression revealed in this qualitative study provide valuable insights into the multimorbid patient perspective on depression. Participants talked about their sense of identity as challenged by the loss of their normal life, including work or family roles, activities or physical function. Consequently, it is not surprising that multimorbid patient interpretation of their symptoms and views about depression often conflicted with sense of identity, which created barriers to acceptance of diagnosis and treatment. Previous research has emphasized the importance of patient beliefs in the diagnosis and treatment of depression in primary care [23]. Beliefs about depression and expectations about interventions also emerged as an important influence, with one participant’s inference that psychotherapy should have a permanent effect reflecting a conceptualization of depression as a temporary dip in mood, not a chronic condition. Both findings emphasize the importance of GP relationship and communication [15], to ensure that patients have an accurate understanding about depression and realistic expectations about treatment.

Situational attribution of depression also emerged as a strong common theme. A recent systematic review of depression and chronic illness found that participants attribute depression to one or more causes, predominantly external [24]. Multimorbid participants in the current study also described situational depression arising from a life-changing event, reporting physical symptoms, functional limitations and health events as causative and inferring that participants believed depression to result from changes to their circumstances. In alignment with recent models of healthcare burden and patient capacity, this suggests that patients might reach a ‘tipping’ point beyond which they are unable to cope with the burden of ‘illness work’ [25], and is consistent with findings describing patient externalisation of mental illness [26]. In light of the common threads of functional/social role losses leading to ‘situational’ depression, GPs should discuss mood or remain vigilant for depression symptoms where functional and social role losses occur.

Whereas Karasz et. al.’s meta-analysis found that patients who presented their emotional distress as situational were rarely offered anti-depressant medications [27], all patients in the current study reported being offered medication for their circumstantially-attributed depression, suggesting that GPs favoured pharmacological interventions in spite of situational attributions and complex multimorbidities. This raises the possibility that somatic and depression symptom overlap may have led some GPs to pathologise distress as clinical depression [28]. Few patients were referred to psychotherapeutic interventions, with several stating that they would have appreciated a non-pharmacological intervention. This reflects previous findings in which GPs acknowledged the benefit of psychotherapy for situational depression, and recognized that depression amongst multimorbid patients was sometimes situational, but withheld psychotherapy referral because of doubts about its efficacy in older patients [16]. In light of participants’ situational attribution of depression, GPs should consider recommending psychotherapy where multimorbid patients have experienced functional and social role losses. Patients might also benefit from engaging in activities that promote a sense of control. Future research should explore the efficacy of psychotherapeutic intervention amongst older, multimorbid patients.

Several multimorbid patients did not recall having been given a diagnosis of depression, although their medication regimens included antidepressant medications; as a consequence their interviews focused on the development and onset of depression symptoms. During the recruitment process two multimorbid patients with a history of depression refused to participate because they were unaware of the diagnosis and believed themselves to be ineligible. This is especially concerning where medication has been prescribed, as several participants reported accepting medication without knowing its purpose. It might reflect patient and GP prioritisation of physical health over psychological health where multiple chronic conditions are present [29], or be a function of long-term relationships between GPs and patients with multimorbidity where issues are carried over for discussion at subsequent clinic appointments [16]. In light of the detrimental effect of poor mental health on physical wellbeing, and the increased complexity generated by multiple chronic conditions, clear communication about symptoms, diagnoses, treatment and priorities is of utmost clinical importance.

Finally, whilst participants were forthcoming about intimate details of their medical experiences and personal lives, all exhibited signs of discomfort while talking about depression. The current study reinforces that conscious and unconscious stigma regarding depression continues to present a challenge for clinicians attempting to identify and treat depression amongst multimorbid patients [9,30]. Strategies to overcome stigma felt by older multimorbid patients would benefit from addressing perceived stigma, guilt regarding self-regulation of mood, and shame relating to inability to cope as raised in previous studies [14,15,24].

### Limitations

Although diagnosis and treatment of depression occurred outside of the multidisciplinary clinic from which we recruited these participants, referral to the clinic might reflect or generate differences between the study population and patients not referred to such a clinic. Additionally, the participant group was limited to multimorbid patients willing to be interviewed about depression. Participation might reflect greater openness to discussing emotions, increasing the likelihood that depression will be diagnosed by their GP [31]. A personal quality of openness might create participant bias, as less forthcoming patients might be less likely to be diagnosed with depression, and even when diagnosed might be less likely to be willing to participate in research about depression. Further, the study was limited to a small number of participants with a recorded diagnosis of depression, however, saturation of themes was achieved after ten interviews and no new themes arose from two confirmatory interviews. Future studies could gain further understanding of the barriers to diagnosis by identifying participants living with depression symptoms that do not have a recorded diagnosis.

## Conclusions

Multimorbid patients attributed depression to life-changing events, many of which were related to their physical health and resulted in threats or changes to their sense of identity. Beliefs about depression, about themselves, and about symptom causation and treatment efficacy strongly affected the diagnostic and treatment process. Functional and social role losses present a clear context in which GPs should broach mood, with the situational attribution of depression suggesting that psychotherapy, which is rarely offered, may be beneficial in these circumstances.

## Competing interests

The authors declared no potential conflicts of interest with respect to the research, authorship, and/or publication of this article.

## Authors’ contributions

MS conceptualized the study, recruited and interviewed participants, performed data analysis and interpretation, and drafted the manuscript. CB assisted with the design of the study, data analysis and interpretation, and drafting the manuscript. SS facilitated data acquisition and assisted with drafting the manuscript. HR assisted with data interpretation and drafting the manuscript. All authors read and approved the final manuscript.

## Pre-publication history

The pre-publication history for this paper can be accessed here:

http://www.biomedcentral.com/1471-2296/15/124/prepub

## References

[B1] Borus JF Howes MJ Devins NP Rosenberg R Livingston WW Primary health care providers’ recognition and diagnosis of mental disorders in their patients Gen Hosp Psychiatry 1988 10 5 317 321 10.1016/0163-8343(88)90002-3 3169532

[B2] Kamphuis MH Stegenga BT Zuithoff NP King M Nazareth I de Wit NJ Geerlings MI Recognition Of Depression In Primary Care: Does It Affect Outcome? The Predict-Nl Study Fam Pract 2011 2011/08/24 10.1093/fampra/cmr049 21859837

[B3] Mitchell AJ Rao S Vaze A Can general practitioners identify people with distress and mild depression? a meta-analysis of clinical accuracy J Affect Disord 2011 130 1–2 26 36 20708274 10.1016/j.jad.2010.07.028

[B4] Stanners M Barton C Shakib S Winefield H The prevalence of depression amongst outpatients with multimorbidity Health 2013 5 4 805 810 10.4236/health.2013.54106

[B5] Mitchell AJ Rao S Vaze A Do primary care physicians have particular difficulty identifying late-life depression? a meta-analysis stratified by age Psychother Psychosom 2010 79 5 285 294 10.1159/000318295 20616623

[B6] Saito M Kawabata H Murakami M Maezawa M Factors in the awareness of depression, focusing on perceptual dissimilarities between PCPs and patients: an exploratory and qualitative research Hokkaido Igaky Zasshi 2011 86 2 79 83 21485321

[B7] Johnston O Kumar K Peveler R Ganbbay J Kendrick T Qualitative study of depression: management in primary care Br J Gen Pract 2007 57 872 879 17976282 10.3399/096016407782318026 PMC2169333

[B8] Fortin M Bravo G Hudon C Lapointe L Dubois MF Almirall J Psychological distress and multimorbidity in primary care Ann Fam Med 2006 4 5 417 422 10.1370/afm.528 17003141 PMC1578652

[B9] Murray J Banerjee S Byng R Tylee A Bhugra D Macdonald A Primary care professionals’ perceptions of depression in older people: a qualitative study Soc Sci Med 2006 63 5 1363 1373 10.1016/j.socscimed.2006.03.037 16698157

[B10] Gunn JM Ayton DR Densley K Pallant JF Chondros P Herrman HE Dowrick CF The association between chronic illness, multimorbidity and depressive symptoms in an Australian primary care cohort Soc Psychiatry Psychiatr Epidemiol 2012 47 2 175 184 10.1007/s00127-010-0330-z 21184214

[B11] Coventry PA Hays R Dickens C Bundy C Garrett C Cherrington A Chew-Graham C Talking about depression: a qualitative study of barriers to managing depression in people with long term conditions in primary care BMC Fam Pract 2011 vol. 12, 2011/03/24 10 10.1186/1471-2296-12-10 PMC3070666 21426542

[B12] Lopez V Copp G Brunton L Molassiotis A Symptom experience in patients with gynecological cancers: the development of symptom cIusters through patient narratives J Support Oncol 2011 9 2 64 71 10.1016/j.suponc.2011.01.005 21542413

[B13] Ryan M Farrelly M Living with an unfixable heart: a qualitative study exploring the experience of living with advanced heart failure Eur J Cardiovasc Nurs 2009 8 3 223 231 10.1016/j.ejcnurse.2009.02.005 19297250

[B14] Maxwell M Women’s and doctors’ accounts of their experiences of depression in primary care: the influence of social and moral reasoning on patients’ and doctors’ decisions Chronic Illn 2005 1 1 61 71 10.1177/17423953050010010401 17136934

[B15] Malpass A Shaw A Sharp D Walter F Feder G Ridd M Kessler D “Medication career” or “Moral career”? The two sides of managing antidepressants: A meta-ethnography of patients’ experience of antidepressants Soc Sci Med 2009 68 1 154 168 10.1016/j.socscimed.2008.09.068 19013702

[B16] Stanners MN Barton CA Shakib S Winefield HR A qualitative investigation of the impact of multimorbidity on GP diagnosis and treatment of depression in Australia Aging Ment Health 2012 16 8 1058 1064 10.1080/13607863.2012.702730 22838401

[B17] Kaplan MH Feinstein AR The importance of classifying initial co-morbidity in evaluatin the outcome of diabetes mellitus J Chronic Dis 1974 27 7–8 387 404 4436428 10.1016/0021-9681(74)90017-4

[B18] Luszcz M The Australian Longitudinal Study Of Ageing: 15 Years Of Ageing In South Australia 2007 Adelaide: Flinders University, South Australia xiii, 130

[B19] Charmaz K Denzin NK, Licoln YS Grounded Theory: Objectivist and Constructivist Methods Handbook of Qualitative Research 2000 Thousand Oaks: Sage Publications 509 536

[B20] Corbin J Strauss A Basics of Qualitative Research: Techniques and Procedures for Developing Grounded Theory 2007 3 Thousand Oaks, CA: Sage

[B21] NCH Software Pty Ltd Express Scribe Transcription Software 2009 513

[B22] QSR International Pty Ltd Nvivo Qualitative Data Analysis Software 2010 9

[B23] Hansson M Chotai J Bodlund O What made me feel better? Patients’ own explanations for the improvement of their depression Nord J Psychiatry 2012 2012/01/04 10.3109/08039488.2011.644807 22211274

[B24] Alderson SL Foy R Glidewell L McLintock K House A How patients understand depression associated with chronic physical disease--a systematic review BMC Fam Pract 2012 vol. 13, 2012/05/30 41 10.1186/1471-2296-13-41 PMC3439302 22640234

[B25] Tanisha J Laurann Y Paul Mathews W Time spent on health related activities associated with chronic illness: a scoping literature review BMC Public Health 2012 vol. 12 10.1186/1471-2458-12-1044 PMC3533987 23206340

[B26] Burton C McGorm K Weller D Sharpe M The interpretation of low mood and worry by high users of secondary care with medically unexplained symptoms BMC Fam Pract 2011 12 1 107 10.1186/1471-2296-12-107 21961785 PMC3197491

[B27] Karasz A Dowrick C Byng R Buszewicz M Ferri L Olde Hartman TC van Dulmen S van Weel-Baumgarten E Reeve J What we talk about when we talk about depression: doctor-patient conversations and treatment decision outcomes Br J Gen Pract 2012 62 594 e55 63 10.3399/bjgp12X616373 22520683 PMC3252540

[B28] Delisle VC Beck AT Ziegelstein RC Thombs BD Symptoms of heart disease or its treatment may increase Beck Depression Inventory Scores in hospitalized post-myocardial infarction patients J Psychosom Res 2012 73 3 157 162 10.1016/j.jpsychores.2012.07.001 22850253

[B29] Munson M Proctor E Morrow-Howell N Fedoravicius N Ware N Case managers speak out: responding to depression in community long-term care Psychiatr Serv 2007 58 8 1124 1127 10.1176/appi.ps.58.8.1124 17664526

[B30] Docherty JP Barriers to the diagnosis of depression in primary care J Clin Psychiatry 1997 58 Suppl 1 5 10 9054902

[B31] Walters P Tylee A Mood disorders in primary care Psychiatry 2006 5 4 138 141 10.1383/psyt.2006.5.4.138 17288504

